# Ascorbic acid stimulates the *in vitro* myoblast proliferation and migration of pacu (*Piaractus mesopotamicus*)

**DOI:** 10.1038/s41598-019-38536-4

**Published:** 2019-02-18

**Authors:** Bruno Oliveira Silva Duran, Guilherme Alcarás Góes, Bruna Tereza Thomazini Zanella, Paula Paccielli Freire, Jessica Silvino Valente, Rondinelle Artur Simões Salomão, Ana Fernandes, Edson Assunção Mareco, Robson Francisco Carvalho, Maeli Dal-Pai-Silva

**Affiliations:** 1 São Paulo State University (UNESP), Institute of Biosciences, Department of Morphology, Botucatu, São Paulo Brazil; 2 University of West São Paulo (UNOESTE), Presidente Prudente, São Paulo Brazil; 3 São Paulo State University (UNESP), Institute of Biosciences, Department of Chemistry and Biochemistry, Botucatu, São Paulo Brazil

## Abstract

The postembryonic growth of skeletal muscle in teleost fish involves myoblast proliferation, migration and differentiation, encompassing the main events of embryonic myogenesis. Ascorbic acid plays important cellular and biochemical roles as an antioxidant and contributes to the proper collagen biosynthesis necessary for the structure of connective and bone tissues. However, whether ascorbic acid can directly influence the mechanisms of fish myogenesis and skeletal muscle growth remains unclear. The aim of our work was to evaluate the effects of ascorbic acid supplementation on the *in vitro* myoblast proliferation and migration of pacu (*Piaractus mesopotamicus*). To provide insight into the potential antioxidant role of ascorbic acid, we also treated myoblasts *in vitro* with menadione, which is a powerful oxidant. Our results show that ascorbic acid-supplemented myoblasts exhibit increased proliferation and migration and are protected against the oxidative stress caused by menadione. In addition, ascorbic acid increased the activity of the antioxidant enzyme superoxide dismutase and the expression of *myog* and *mtor*, which are molecular markers related to skeletal muscle myogenesis and protein synthesis, respectively. This work reveals a direct influence of ascorbic acid on the mechanisms of pacu myogenesis and highlights the potential use of ascorbic acid for stimulating fish skeletal muscle growth.

## Introduction

*Piaractus mesopotamicus*, which is popularly known as pacu, is a teleost fish with major economic importance and a high market value in Brazilian fisheries and pisciculture. The distribution of pacu is highly concentrated in wetland areas in the Midwest Brazilian region^1^ and, along with other members of the Characidae family (*Piaractus mesopotamicus*, *Colossoma macropomum*, *Piaractus brachypomus* and hybrids), pacu represents most native fish farmed, accounting for almost 40% of the national production^2^. In addition to its high economic value, the use of pacu in scientific research has recently increased. Zebrafish (*Danio rerio*) and medaka (*Oryzias latipes*) are teleost fish extensively used for research in many fields, such as genetics, embryology, physiology, toxicology and nutrition, but there are some limitations due to their small sizes, such as the study of myogenesis in cell cultures^3^. Because pacus have a large body size, they have the potential to serve as an excellent model for studies investigating muscle growth in fish.

Skeletal muscle is the most abundant tissue in teleost fish and constitutes approximately 60% of the total body mass. Skeletal muscle allows underwater propulsion, represents the main protein reservoir in fish and forms the bulk of the fillet, which is the main product in the aquaculture industry^4,5^. Postembryonic muscle growth in fish involves a population of resident myogenic precursor cells and encompasses the main events of embryonic myogenesis^6^. Once activated, these cells give rise to myoblasts, whose proliferation, migration and differentiation are the mechanisms that promote hyperplasia (increase in muscle fiber number) and hypertrophy (increase in muscle fiber size)^6,7^.

In such a context, fish myoblast cell culture represents a very useful *in vitro* tool to obtain an understanding of the regulation of muscle growth and myogenesis^6,8–10^. By recapitulating key steps, such as cell proliferation and differentiation, myoblast cell culture provides a controlled environment for studying myogenesis regulation^10,11^. Similarly, cell culture media can be modified to evaluate the role of nutrients, growth factors and drugs under precisely controlled conditions^6,9^. Our research group has been successful in standardizing pacu myoblast cell culture, providing a great advance in the understanding of muscle plasticity in this species and generating an important tool for fish muscle growth research^12^.

Food intake, composition and availability represent important factors leading to muscle growth^6^. In general, fish exhibit mammal-like nutritional requirements for growth, reproduction and other physiological functions, and in confinement, fish require a nutritionally complete and balanced diet^13^. Several studies have shown that ascorbic acid (vitamin C) plays an important role in the diet of fish. Ascorbic acid-deficient diets, especially fed to larvae fish^14^, promote reduced growth, impaired feed conversion, skeletal deformities in the operculum and cartilage of the gills, anemia, delay or decrease in wound healing, reduction in reproductive performance and decrease in hatchability^13,15,16^. Ascorbic acid plays several important cellular and biochemical roles as an antioxidant because of its high reducing potential^17^. Ascorbic acid neutralizes reactive oxygen species (ROS) produced during cellular metabolism or functional activities, which have deleterious effects on several molecules in excessive amounts (oxidative stress)^18^. Oxidative stress can be induced chemically using stressing agents, such as menadione (2-methyl-1,4-naphtoquinone)^19^. Menadione is a polycyclic aromatic ketone that has been widely used as an oxidant and has demonstrated cytotoxic activity via the elevation of superoxide anions and hydrogen peroxide^19–21^. As a cellular reducing agent, ascorbic acid also plays a role in collagen biosynthesis, acting as a cofactor in the hydroxylation of lysine and proline present in procollagen^17^. The formation of a stable collagen matrix is crucial for the structure and maintenance of connective tissue, stimulation of osteogenesis and bone growth^22,23^. Therefore, ascorbic acid directly influences the growth of animals, including fish species, and is necessary for the normal development of their bodies^13^. However, whether ascorbic acid influences fish growth exclusively due to its action on connective and bone tissues or whether it can directly influence the mechanisms of skeletal muscle growth remain unclear.

In skeletal muscle, ascorbic acid is a key factor enhancing carnitine biosynthesis^24^, which plays an important role in energy production via beta-oxidation. In addition, this vitamin facilitates glycogen storage^25^ and protects cells against exercise-induced ROS generation^26,27^. Muscle tissues contain 40% of the whole-body ascorbic acid content^28^. Some studies have shown that ascorbic acid plays a role in myogenesis *in vitro*, but these studies have focused on myoblast differentiation into myotubes. MacBride (1989) showed the early fusion of chicken embryo myoblasts after the addition of ascorbic acid^29^. Moreover, ascorbic acid could increase the expression of myogenin, which is a differentiation-related myogenic factor, in myoblasts incubated at both 37 °C^30^ and 30 °C^31^. Ikeda *et al*. (2017) observed that ascorbic acid supplementation in C2C12 myoblasts enhanced myotube formation (differentiation rate); although this ascorbic acid supplementation had no effect on myotube hypertrophy^32^. Compared to myotubes, myoblasts have an increased ability to transport ascorbic acid and exhibit increased DHA (*dehydroascorbic acid*) reductase activity necessary for ascorbic acid metabolic recycling^28^. This same study showed that L6 fast myoblasts^33^ are more efficient in ascorbic acid import than C2C12 myoblasts^28^. However, to the best of our knowledge, no study has investigated the role of ascorbic acid in *in vitro* fish myoblast proliferation and migration.

Because fish are highly susceptible to ascorbic acid-deficient diets during the early stages of growth^13,14^, we hypothesized that ascorbic acid also has a direct influence on early muscle growth in fish. Thus, the aim of our work was to evaluate myoblast proliferation and migration, which are processes related to the onset of myogenesis, in pacu myoblast cell cultures supplemented with ascorbic acid and its antioxidant role against menadione.

## Results

### Myoblast cell culture

**Figure 1:**
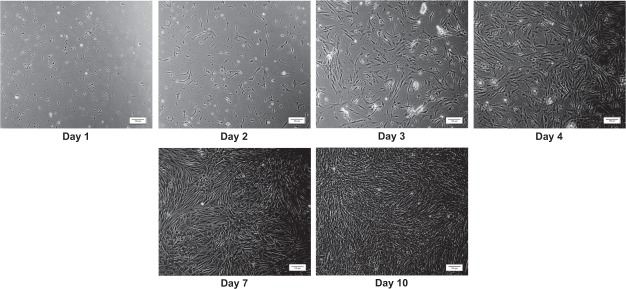
Myoblast cell cultures established using fast-twitch skeletal muscle from juvenile pacus. Myoblasts on days 1 to 4 (during this period, the proliferation and migration mechanisms were studied) and days 7 and 10 (the stages of myotube formation) of cell culture. The images were obtained under an inverted microscope at a 10x magnification (Bars: 100 µm).

We successfully isolated and established myoblast cell cultures from the fast-twitch muscle of pacu. The myoblasts showed normal development as reported in a previous work that published results of pacu myoblast cell cultures^12^. However, to increase the amount of RNA and protein for the proposed analyses, we seeded the cells at a high concentration (3 × 10^6^ cells/mL), which reduced the time until the formation of myotubes. We observed an initial stage of round mononucleated cells on days 1-2, their progressive proliferation and elongation between days 3–6, and a final stage of myotubes formation on days 7–10 (Fig. 1). We chose to study myoblast proliferation and migration within 4 days after cell plating, since during this time the cells reached 100% confluency and began to fuse and differentiate into myotubes. The myoblasts were separated into the following 4 experimental groups: nontreated myoblasts (CTR group), menadione treated myoblasts (MEN group), ascorbic acid supplemented myoblasts (AA group), and menadione plus ascorbic acid treated myoblasts (MEN + AA group). In addition to the expression of myogenic regulatory factors (MRFs) already published in our previous work^12^, the myogenic cells were identified by staining for desmin, and the nuclei were counterstained with DAPI (4,6-diamidino-2-phenylindole) (Supplementary Fig. S1).

We initially performed trypan blue exclusion and MTT (Thiazolyl Blue Tetrazolium Bromide - Sigma-Aldrich, USA) assays to verify the ideal menadione concentration and treatment duration that causes oxidative stress but maintains cell viability (Supplementary Fig. S2). The trypan blue exclusion assay showed that menadione treatment for 24 hours drastically reduced myoblast viability, and therefore, this incubation time was quickly discarded from consideration for our studies. Compared with the myoblasts treated only with phosphate buffered saline (PBS), the 1-hour menadione treatment reduced the myoblast viability by 6% (0.1 µM), which was not sufficient to promote oxidative stress, 21% (1 and 10 µM) and 26% (100 µM) (Supplementary Fig. S2A). Similarly, the MTT assay showed that the higher menadione concentrations significantly decreased myoblast viability (Supplementary Fig. S2B). We chose the 10 μM concentration of menadione and 1-hour duration for the myoblast treatments.

### Myoblast proliferation assay

**Figure 2:**
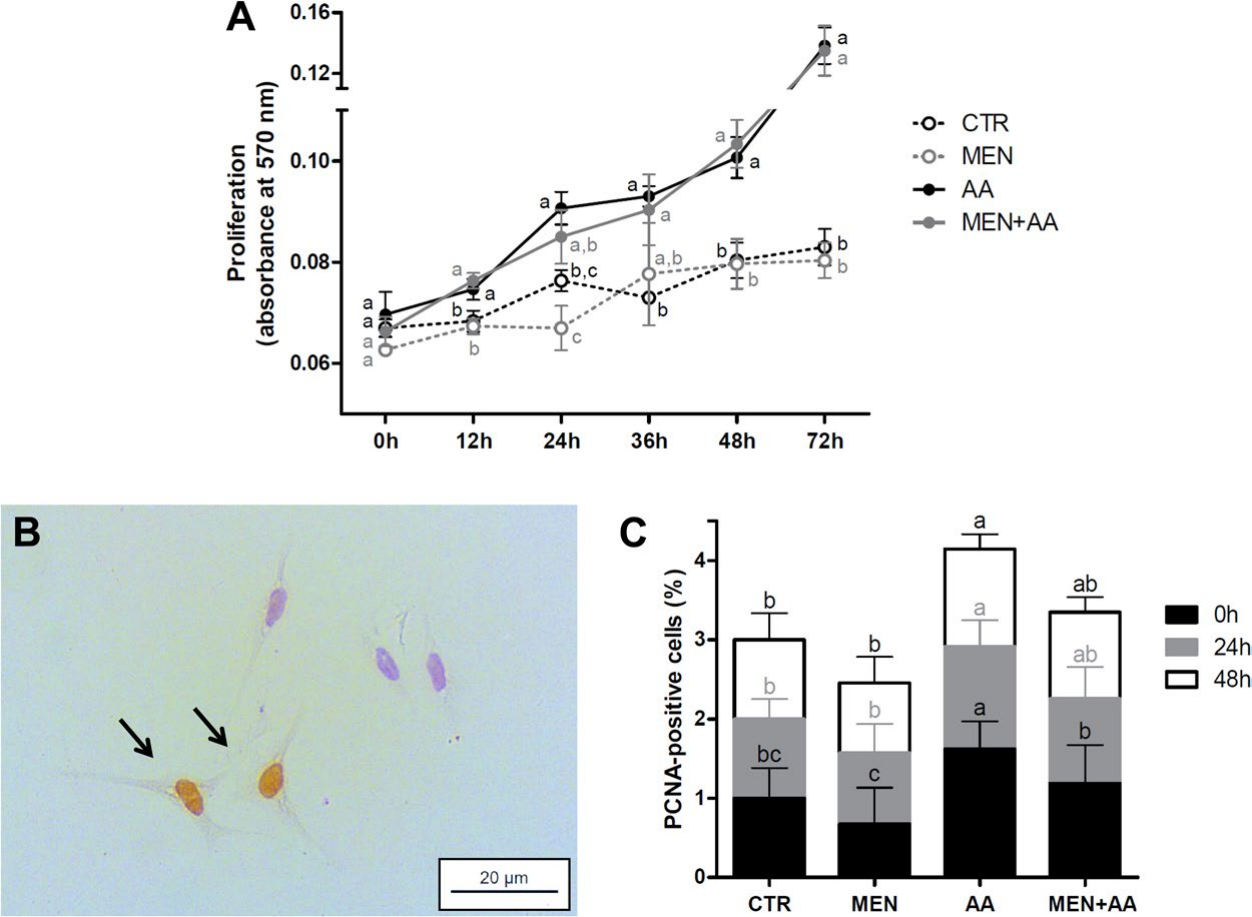
Myoblast proliferation. Cell proliferation was evaluated in myoblasts incubated with menadione (MEN), ascorbic acid (AA), menadione combined with ascorbic acid (MEN + AA) or without treatment (CTR). Ascorbic acid was administered at 200 μM to AA and MEN + AA myoblasts, and menadione was administered at 10 µM to MEN and MEN + AA myoblasts. (**A**) MTT assay. Myoblast proliferation was measured considering the absorbance variation 0, 12, 24, 36, 48 and 72 hours after day 1. The data are expressed as the absorbance at 570 nm and presented as the mean ± SD of duplicates from three independent cell cultures. (**B**) PCNA immunostaining. Representative image of PCNA immunostaining in myoblasts in the CTR group on day 1. Arrows indicate PCNA-positive myoblasts. Image was obtained under a light microscope at a 40x magnification (Bars: 20 µm). (**C**) Quantification of PCNA-positive cells. Myoblast proliferation was measured considering the percentage of PCNA-stained nuclei 0, 24 and 48 hours after day 1. The data are expressed as the fold change compared with the CTR group and presented as the mean ± SD of duplicates from three independent cell cultures. The different letters indicate significant differences among the groups (p < 0.05 - One-way ANOVA test, followed by Tukey’s multiple comparisons test).

Compared to the CTR and MEN groups, the ascorbic acid supplementation significantly stimulated myoblast proliferation based on both the MTT assay (Fig. 2A) and PCNA (*proliferating cell nuclear antigen*) immunostaining (Fig. 2B,C). The MTT assay indicated that the proliferation of the ascorbic acid-supplemented myoblasts was increased with significant differences after 12 hours (p < 0.05). The positive effect of ascorbic acid in the MEN + AA group overcame the menadione oxidative effects, and these myoblasts exhibited a proliferative rate similar to that observed in the AA group at all evaluated time points (Fig. 2A). The PCNA immunostaining showed significantly increased proliferation in the AA group on the day following cell plating (0 h) (p < 0.01) and similar proliferation between the AA and MEN + AA groups at 24 and 48 hours, which was statistically higher that in the CTR and MEN myoblasts (p < 0.05) (Fig. 2C). No significant differences were found between the CTR and MEN groups (p > 0.05) (Fig. 2A,C).

### Myoblast migration assay

**Figure 3:**
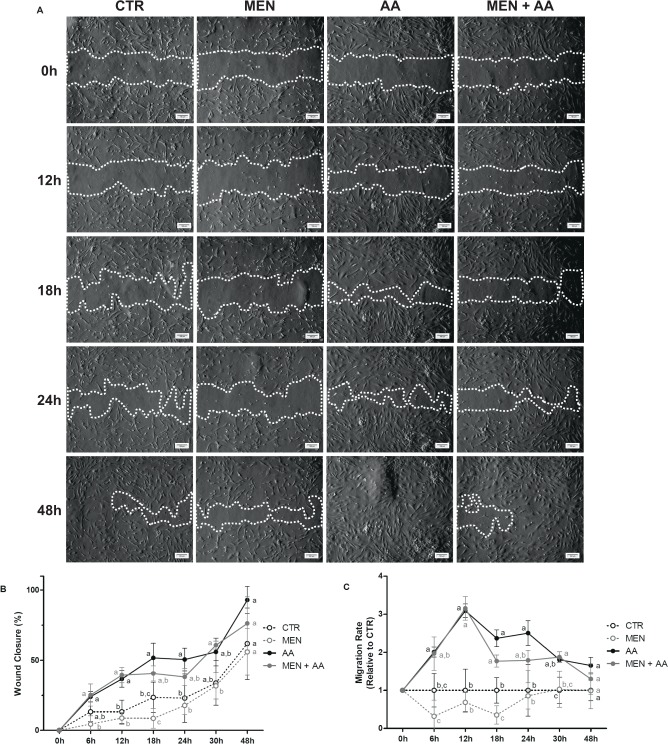
Myoblast migration. Cell migration was evaluated in myoblasts incubated with menadione (MEN), ascorbic acid (AA), menadione combined with ascorbic acid (MEN + AA) or without treatment (CTR). Ascorbic acid was administered at 200 μM to AA and MEN + AA myoblasts, and menadione was administered at 10 µM to MEN and MEN + AA myoblasts. Myoblast migration was measured 0, 6, 12, 18, 24, 30 and 48 hours postwound. (**A**) Representative images of wound healing assay. The white dashed line delimits the wound area. The images were obtained under an inverted microscope at a 10x magnification (Bars: 100 µm). (**B**) Wound closure (reduction in wound area sizes over time). The data are expressed as a percentage and presented as the mean ± SD of duplicates from three independent cell cultures. (**C**) Migration rate (variation in wound closure over a time interval). The data are expressed as the fold change compared with the CTR group and presented as the mean ± SD of duplicates from three independent cell cultures. The different letters indicate significant differences among the groups (p < 0.05 - One-way ANOVA test, followed by Tukey’s multiple comparisons test).

Considering wound closure, similar myoblasts migration was observed between the AA and MEN + AA groups and between the CTR and MEN groups (Fig. 3B). The AA myoblasts showed increased migration compared to those in the CTR and MEN groups at 6, 12, 18 and 24 hours (p < 0.05). At these initial time points, the MEN myoblasts showed the lowest migration rate, whereas the ascorbic acid-supplemented myoblasts showed the highest migration rate at 12 hours (Fig. 3C). The comparison between the MEN and MEN + AA groups, specifically at 6, 12, 18 and 30 hours (p < 0.05), demonstrated that the ascorbic acid supplementation could reduce menadione’s effects and increase migration in the MEN + AA myoblasts. At 48 hours, although no significant differences were observed (p = 0.1453), the AA myoblasts were able to completely fill the wound area, while some spaces were still visible in the other groups (Fig. 3A,B).

### Superoxide dismutase and catalase activities

**Figure 4:**
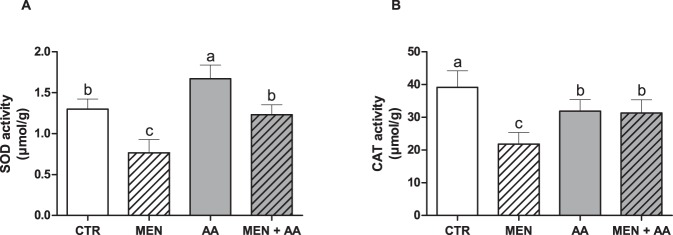
Activities of the antioxidant enzymes superoxide dismutase (SOD) and catalase (CAT). The activities of the SOD (**A**) and CAT (**B**) enzymes were evaluated in myoblasts incubated with menadione (MEN), ascorbic acid (AA), menadione combined with ascorbic acid (MEN + AA) or without treatment (CTR). Ascorbic acid was administered at 200 μM to AA and MEN + AA myoblasts, and menadione was administered at 10 µM to MEN and MEN + AA myoblasts. The data are expressed as µmol per g of protein and presented as the mean ± SD of duplicates from three independent cell cultures. The different letters indicate significant differences among the groups (p < 0.05 - One-way ANOVA test, followed by Tukey’s multiple comparisons test).

The superoxide dismutase (SOD) and catalase (CAT) activities in the MEN group were significantly lower than those in the CTR group (p < 0.001). Both the AA and MEN + AA groups had increased SOD and CAT activities compared to those in the MEN group (p < 0.01). Ascorbic acid also resulted in significantly higher SOD activity in the myoblasts in the AA group compared with that in the CTR group (p < 0.01) (Fig. 4).

### mRNA expression

Given the high density of the cells seeded on the cell culture plates (3 × 10^6^ cells/mL), we evaluated the expression profile of typical muscle genes throughout development, especially on day 4, when the experiment was performed. On day 4, the myoblasts expressed high levels of all genes evaluated, showing that ascorbic acid experimentation could be properly conducted at this stage of proliferation (Supplementary Fig. S3).

**Figure 5:**
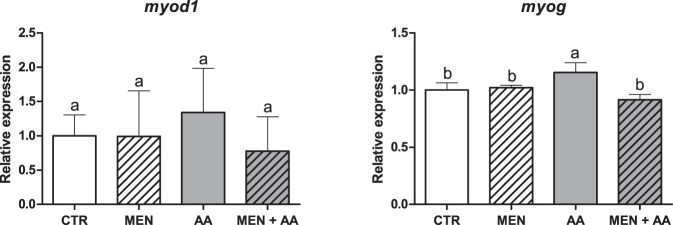
Relative mRNA expression of *myod1* and *myog*. *myod1* and *myog* mRNA expression was assessed by qPCR in myoblasts incubated with menadione (MEN), ascorbic acid (AA), menadione combined with ascorbic acid (MEN + AA) or without treatment (CTR). Ascorbic acid was administered at 200 μM to AA and MEN + AA myoblasts, and menadione was administered at 10 µM to MEN and MEN + AA myoblasts. The data are expressed as the fold change compared with the CTR group and presented as the mean ± SD of duplicates from four independent cell cultures. The different letters indicate significant differences among the groups (p < 0.05 - One-way ANOVA test, followed by Tukey’s multiple comparisons test).

**Figure 6:**
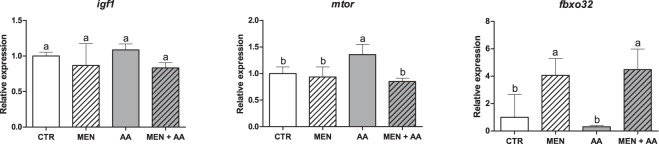
Relative mRNA expression of *igf1*, *mtor* and *fbxo32*. *igf1*, *mtor* and *fbxo32* mRNA expression was assessed by qPCR in myoblasts incubated with menadione (MEN), ascorbic acid (AA), menadione combined with ascorbic acid (MEN + AA) or without treatment (CTR). Ascorbic acid was administered at 200 μM to AA and MEN + AA myoblasts, and menadione was administered at 10 µM to MEN and MEN + AA myoblasts. The data are expressed as the fold change compared with the CTR group and presented as the mean ± SD of duplicates from four independent cell cultures. The different letters indicate significant differences among the groups (p < 0.05 - One-way ANOVA test, followed by Tukey’s multiple comparisons test).

The differences in the expression of *myod1* (*myogenic differentiation 1*) among the groups were not statistically significant (p = 0.5607), whereas the expression of *myog* (*myogenin*) was slightly upregulated in the AA group compared to that in the CTR, MEN and MEN + AA groups (p < 0.05) (Fig. 5). Furthermore, the treatment of the myoblasts according to their experimental groups did not promote significant differences in *igf1* (*insulin-like growth factor 1*) expression (p = 0.1644), but the *mtor* (*mechanistic target of rapamycin*) levels were significantly upregulated in the AA group (p < 0.05). In contrast, the expression levels of *fbxo32* (*f-box protein 32*) were significantly upregulated in the MEN and MEN + AA groups compared to those in the CTR and AA groups (p < 0.05) (Fig. 6).

## Discussion

### Myoblast proliferation and migration

Ascorbic acid supplementation in the fish cell cultures increased myoblast proliferation and migration. Even the myoblasts in the MEN + AA group exhibited an enhancement in these mechanisms, especially compared to the MEN group, highlighting the ability of ascorbic acid to suppress the harmful effects of menadione.

Cell proliferation and migration are essential steps in myogenesis and muscle growth^6^. The increased myoblast proliferation and migration induced by ascorbic acid started early in our experiment and was observed within 1 day of the experiment with a gradual increase in these mechanisms over time. In addition, fish embryos initiate myogenesis during an earlier stage of development than amniotes, which probably reflects the requirement for the generation of swimming propulsion during earlier life stages^6^. This earlier myogenesis and the anticipated action of ascorbic acid help explain the high susceptibility of fish during the early stages of growth to ascorbic acid-deficient diets^13,14^, which could lead to impaired myogenesis in addition to connective and bone tissue deformities. We believe that the myoblast proliferation and migration periods may be crucial for the use of ascorbic acid and other interventions, such as amino acids and endocrine factors, aiming to increase skeletal muscle growth.

In fact, several studies have investigated myogenesis regulation by Igfs, growth hormone (Gh) and amino acids and provided many indications of their proliferative roles. The proliferation of sea bream (*Sparus aurata*) myoblasts was significantly stimulated by Igf1, Igf2 and Gh treatments^34,35^. Moreover, Gh and Igf2 increased the expression of *myf5* (*myogenic factor 5*) and *myod2*^35^, which are primary MRFs involved in the initial steps of myogenesis, mainly determination, proliferation and migration^36–38^. Amino acid treatments revealed an interplay with Igfs in Atlantic salmon (*Salmo salar*) myogenic cells, promoting increased expression levels of Igf signaling components^8^ and *myod1c*^39^ on day 6 of development. This role in proliferation was also observed in a study investigating the impact of amino acid deficiencies on sea bream myoblasts, in which Lysine limitation significantly decreased the mRNA expression of Igf signaling components, *myf5* and *myod2*^40^.

In our work, it is possible that ascorbic acid increased myoblast proliferation and migration through its role as an antioxidant agent that could decrease the levels of ROS within the cell. Increased or decreased levels of ROS in the cytoplasm lead to alterations in the redox status of the cell, which is important for controlling the activation of many factors^41,42^. In fact, Barbieri and Sestili (2012) discussed the most relevant signaling pathways triggered and/or affected by ROS in skeletal muscle, with low levels of ROS inducing upregulation of Igf1 and Pgc-1α (*peroxisome proliferator activated receptor gamma coactivator 1 alpha*), and high levels of ROS upregulating FoxO (*forkhead box O*) or Nf-κB (*nuclear factor kappa B*) signaling pathways^43^. In our work, the increased proliferation and migration induced by ascorbic acid were probably due to its direct regulation of the redox status, able to decrease ROS levels/persistence in myoblasts and, consequently, modulate components of the Igf signaling pathway. Moreover, given the crosstalk among signaling pathways controlling muscle growth^44,45^, the redox status could also regulate the expression or activity of factors related to myogenesis, such as MRFs. A relatively recent study showed the effects of other antioxidants on primary myogenic cells isolated from skeletal muscle of rainbow trout, testing different doses of anthocyanidins^46^. The anthocyanidins upregulated the expression of *gpx1* (*glutathione peroxidase 1*) and *pax7* (*paired box protein 7*), which contribute to enhancing muscle tissue defense against oxidative stress and delay the progress of myogenic differentiation, respectively^46^, maintaining the cells in a quiescent or proliferative state.

### Superoxide dismutase and catalase activities

Although no direct measure of ROS were used to confirm the occurrence of menadione-induced oxidative stress and the antioxidant role of ascorbic acid, we evaluated the activities of the antioxidant enzymes SOD and CAT. The myoblasts treated with menadione showed a significant reduction in both SOD and CAT activities, whereas the ascorbic acid supplementation in the AA and MEN + AA groups maintained the high activity of these antioxidant enzymes compared to that in the MEN group. SOD and CAT act sequentially to neutralize oxidative stress. SOD dismutates superoxide anion radicals (O_2_^−^) generated in the mitochondrial electron transport chain into hydrogen peroxide (H_2_O_2_), which is converted into H_2_O by CAT^47,48^. Due to the high oxidative potential of menadione, we believe that ROS produced following the menadione treatment may have promoted extensive cellular damage and that the antioxidant system could not resist this oxidant-induced injury. However, ascorbic acid, which was continuously supplemented in the cell cultures, may have stimulated the myoblast antioxidant machinery in preparation for the eventual oxidative stress, especially by increasing the activity of SOD, which could act first to avoid exacerbated increases in the ROS levels. In addition, the maintained activities of SOD and CAT by ascorbic acid prevented the drastic oxidative stress caused by menadione in the MEN + AA group.

Similar to our results, Khassaf *et al*. (2003) showed that the baseline activities of both SOD and CAT were elevated in lymphocytes from ascorbic acid-supplemented individuals^49^. Lü *et al*. (2010) proposed that antioxidants may act through the enhancement of the activity or expression of antioxidant enzymes, such as SOD, CAT and glutathione peroxidase^18^. In fact, the antioxidant-induced activation of these internal antioxidant enzymes has been studied in different cell line models. Treatment with anthocyanins promoted positive effects on elevating the antioxidant capacity, including increasing the activities of glutathione reductase and peroxidase, in rat hepatocytes^50^. Robb *et al*. (2008) investigated the long-term exposure of human fibroblasts to resveratrol and observed a dramatic and progressive upregulation of mitochondrial SOD expression and activity^51^. This activation of intracellular antioxidant enzymes by an external antioxidant could explain the high SOD activity in the fish myoblasts supplemented with ascorbic acid in our study. Our results confirm that menadione induced oxidative stress and that ascorbic acid plays an antioxidant role.

### mRNA expression

Since skeletal muscle plasticity and individual fiber growth result from a balance between protein synthesis and degradation^42,52^, to strengthen our work, we evaluated the mRNA expression of some molecular markers of myogenesis (*myod1* and *myog*), protein synthesis (*igf1* and *mtor*) and protein degradation (*fbxo32*) in pacu myoblasts.

The MRFs Myod1 and Myog control the myogenic program inside the cell and constitute the key factors that determine the progression of myogenesis^53^. In our study, the significant increase in the *myog* levels in the ascorbic acid-supplemented myoblasts represents an enhanced differentiation mechanism. This increase probably occurred due to the increased proliferation and migration of the myoblasts in the AA group as observed in the proliferation and migration assays despite the absence of significant differences in the expression levels of *myod1*. Since proliferation and migration were elevated, the myoblasts could express higher levels of *myog* and initiate fusion and differentiation earlier. Mitsumoto *et al*. (1994) showed that rat L6 myoblasts expressed higher amount of myogenin at both the mRNA and protein levels after treatment with ascorbic acid^30^, and Shima *et al*. (2011) observed that ascorbic acid increased myogenin expression in mouse C2C12 myoblasts incubated at 30 °C, promoting myogenic differentiation at low temperatures^31^. Based on these findings, we can infer that ascorbic acid supplementation is able to accelerate and advance the beginning of myogenesis in fish myoblasts.

Igf1, which is among the most studied and best characterized muscle growth-promoting factors, triggers several downstream cascades that culminate with the activation of Mtor and other components^6,52^. Mtor processes and integrates signals from nutrients, energy status and growth factors, controlling protein synthesis among its other functions^54^. Although *igf1* expression did not significantly differ among the experimental groups, *mtor* was highly expressed in the myoblasts treated exclusively with ascorbic acid, which is indicative of increased protein synthesis in the AA group. These results show possible Mtor activation independent of Igf1. Early studies investigating fish myoblast cell cultures have shown that amino acids independently stimulate protein synthesis^54,55^, whereas Igf only stimulates protein synthesis if amino acids are also present in the media^8,9^. These works support the role of nutritional stimulation in skeletal muscle growth in teleost fish, and the activation of *mtor* by our ascorbic acid supplementation could be related to this nutritional pathway, increasing protein synthesis in addition to myoblast proliferation and migration.

Fbxo32, which is also known as Mafbx (*muscle atrophy f-box protein*), regulates the protein degradation in skeletal muscle and presents an usual upregulation during muscle atrophy^52^. The upregulated expression of *fbxo32* in the MEN and MEN + AA groups indicates increased protein degradation in myoblasts probably due to menadione’s action as an oxidant. Similar results were observed by Suzuki *et al*. (2007), who investigated the involvement of the dystrophin glycoprotein complex (DGC) in the regulation of skeletal muscle atrophy^56^. These authors showed that nNOS (*neuronal nitric oxide synthase*) dissociates from the DGC during atrophy and generates nitric oxide that could induce oxidative stress in skeletal muscle. Nitric oxide increased the levels of Fbxo32/Mafbx and other markers related to the protein degradation pathway^56^. In addition, Eijkelenboom and Burgering (2013) reviewed the function of FoxO, which is a well-known upstream activator of Fbxo32, including its increased transcriptional activity following cellular stress and high generation of ROS^57^. These data corroborate our results, demonstrating that the potent oxidative effect of menadione could increase *fbox 32* expression and, consequently, protein degradation. This finding further explains the reduced myoblast proliferation and migration in the MEN group.

## Conclusions

Ascorbic acid supplementation promotes increased myoblast proliferation and migration in cell cultures established using fast-twitch skeletal muscle from pacu. These results may be related to the antioxidant role of ascorbic acid, which protects myoblasts against the harmful effects of menadione, a powerful oxidant.

In addition to the enhanced proliferation and migration, the higher expression of *myog* and *mtor* showed that the ascorbic acid supplementation accelerated the beginning of myogenesis and increased protein synthesis, respectively, indicating that ascorbic acid can directly influence the mechanisms of fish skeletal muscle growth in addition to its action on connective and bone tissue.

Given the beneficial role of ascorbic acid in myogenesis and increased protein synthesis in skeletal muscle, the supplementation of this vitamin in fish is a potential treatment for increasing muscle growth, enabling interventions and improvements in pisciculture of pacu and other fish species.

## Materials and Methods

### Ethics statement and animals

All experiments and procedures were performed in accordance with the Ethical Principles in Animal Research adopted by the Brazilian College of Animal Experimentation (COBEA). The protocol was approved by the Ethics Committee on Animal Use (protocol number 705) of the Institute of Biosciences, São Paulo State University (UNESP), Botucatu, São Paulo, Brazil. Pacus were obtained from the University of West São Paulo (UNOESTE), Presidente Prudente, São Paulo, Brazil. The fish were farmed at 28 °C under a natural photoperiod (12 light:12 dark) in 0.5 m^3^ storage tanks equipped with a water circulation system. Fast-twitch muscles were collected from juvenile pacus weighing 14.08 ± 5.91 g (mean ± SD; n = 10 per cell culture) to establish the primary myoblast cell cultures. The pacus were euthanized with excess benzocaine (Sigma-Aldrich, USA), at a concentration exceeding 250 mg/L, prior to the collection of the muscle samples.

### Myoblast cell culture

The myoblasts were isolated and cultured according to the protocol described by Fauconneau and Paboeuf (2000)^58^. Previous work was already published using myoblast cell cultures of pacu^12^. Briefly, the fast-twitch muscles were collected from the epaxial region and mechanically dissociated with scalpels. To release the muscle cells, the fragments were enzymatically digested with 0.2% collagenase type I and 0.1% trypsin (Sigma-Aldrich, USA). The cell suspension was filtered in cell strainers (Corning, USA), allowing for the remove of the debris, centrifuged and the cell pellet was resuspended in DMEM media (Dulbecco’s Modified Eagle’s Medium, 9 mM NaHCO_3_ and 20 mM HEPES, pH 7.4 - Sigma-Aldrich, USA) with 1% antibiotics and 10% fetal bovine serum (Sigma-Aldrich, USA). The cells were counted using a hemocytometer (Kasvi, Brazil) and diluted at a concentration of 3 × 10^6^ cells/mL, to increase the amount of RNA and protein for the proposed analyses, especially on day 1 (the next day after cell plating). The amount of cells seeded was 3 × 10^6^ cells/well in 6-well plates (1 mL per well), 1.5 × 10^6^ cells/well in 12-well plates (0.5 mL per well) or 0.187 × 10^6^ cells/well in 96-well plates (0.0625 mL per well). DMEM media with 1% antibiotics and 10% fetal bovine serum were added to each well at the same volume of cell suspension (1 mL/well in 6-well plates, 0.5 mL/well in 12-well plates and 0.0625 mL/well in 96-well plates). The cells were plated in wells previously treated with poly-L-lysine and laminin (Sigma-Aldrich, USA), which have high affinity for the myoblasts, and incubated at 28 °C for 10 days. The media were changed once a day and the myoblasts morphology was regularly monitored in inverted microscope (Zeiss, Germany). The results were achieved from three or four independent cell cultures.

### Immunofluorescence

Immunofluorescence was performed in myoblast cell cultures at days 1, 3 and 7 of development in coverslips in 6-well plates. The myoblasts were washed with PBS and then fixed in 4% paraformaldehyde for 15 minutes. The cells were permeabilized with 0.1% Triton X-100 (Sigma-Aldrich, USA) for 10 minutes and incubated in blocking solution (1% glycine, 3% bovine serum albumin (BSA), 8% fetal bovine serum and 0.3% Triton X-100 - Sigma-Aldrich, USA) for 1 hour, to prevent nonspecific binding. The myoblasts were incubated overnight at 4 °C with a rabbit anti-desmin primary antibody (Sigma-Aldrich, USA) diluted in blocking solution (1:20). The cells were washed and then incubated for 2 hours at 4 °C with an anti-rabbit FITC secondary antibody (sc-2090 - Santa Cruz, USA) diluted in blocking solution (1:400). The myoblasts nuclei were counterstained with DAPI present in Vectashield® mounting medium (Vector Laboratories Inc., USA) and the images were acquired under a fluorescence microscope (Olympus, Japan).

### Experimental design and myoblast viability

The following four experimental groups were used to evaluate the role of ascorbic acid in proliferation and migration: the Control group (CTR), in which the myoblasts were not exposed to any treatment; the Menadione group (MEN), in which the myoblasts were treated with menadione (Sigma-Aldrich, USA) to induce oxidative stress; the Ascorbic acid group (AA), in which the myoblasts were supplemented with L-ascorbic acid 2-phosphate (Sigma-Aldrich, USA), which is a stable form of ascorbic acid recommended for use in cell cultures^31,59^; and the Menadione and Ascorbic acid group (MEN + AA), in which the myoblasts were treated with menadione combined with L-ascorbic acid 2-phosphate supplementation to verify the antioxidant effects of ascorbic acid. Based on previous work^31,32,60^, L-ascorbic acid 2-phosphate was added to the DMEM media at a concentration of 200 μM in the AA and MEN + AA groups and was continuously administered to the myoblasts (each time the media were changed) since cell plating. Tests were performed to verify the ideal concentration of menadione causing oxidative stress while maintaining cell viability.

The myoblast viability following the treatment with menadione was accessed by trypan blue exclusion and MTT assays. The trypan blue exclusion assay was performed on day 1 in 6-well plates using duplicates from four independent cell cultures. Menadione was solubilized in dimethyl sulfoxide (DMSO) at 1 mg/mL (stock solution) according to the manufacturer’s guidelines, and different concentrations were prepared after dilution in PBS (working solution). The myoblasts were treated with 0.1, 1, 10 and 100 µM menadione for 1 and 24 hours. After washing with PBS and harvesting with 0.25% trypsin, the myoblasts were stained with 0.4% trypan blue solution (Sigma-Aldrich, USA). The stained (dead) and unstained (live) myoblasts were counted using a hemocytometer (Kasvi, Brazil), and cell viability was expressed as a percentage of the total number of cells. The MTT assay was performed on day 1 in 96-well plates using duplicates from four independent cell cultures. Metabolically active cells reduce tetrazolium MTT into intracellular purple formazan, whose quantity is directly proportional to the viable and proliferative cells over time^61^. The myoblasts were treated with 10, 100 and 200 µM menadione for 1 hour and then incubated with tetrazolium MTT solution (Sigma-Aldrich, USA) at 37 °C for 4 hours. The myoblasts were incubated with 200 μL per well of ≥99.9% DMSO (Sigma-Aldrich, USA) to solubilize the intracellular purple formazan, and the absorbance at 570 nm was measured using an Asys Expert Plus Microplate Reader (Biochrom, United Kingdom). Based on the trypan blue exclusion and MTT assays, the MEN and MEN + AA groups were treated with 10 µM menadione for 1 hour immediately before each experiment. The concentration of 10 µM menadione contained 0.1% DMSO, which is not toxic to cells^62^. To account for the possible effects of DMSO on the menadione treatment, the media in the CTR and AA groups also received the nontoxic concentration of 0.1% DMSO.

### Myoblast proliferation assays

Myoblast proliferation was assessed by an MTT assay and PCNA immunostaining. The MTT assay was performed 0, 12, 24, 36, 48 and 72 hours after day 1 in different 96-well plates seeded on the same day using duplicates from three independent cell cultures. Similar to the cell viability test, the myoblasts were incubated with MTT solution (Sigma-Aldrich, USA) at 37 °C for 4 hours. After the tetrazolium MTT reduction, the resulting intracellular purple formazan was solubilized by incubating the myoblasts with 200 μL per well of ≥99.9% DMSO (Sigma-Aldrich, USA). The absorbance at 570 nm was quantified using an Asys Expert Plus Microplate Reader (Biochrom, United Kingdom). PCNA immunostaining was performed 0, 24 and 48 hours after day 1 in coverslips on 12-well plates using duplicates from three independent cell cultures. The myoblasts were washed with PBS and then fixed in 4% paraformaldehyde for 10 minutes. The cells were postfixed in 100% methanol for 10 minutes and incubated in blocking solution (3% BSA – Sigma-Aldrich, USA) for 1 hour to prevent nonspecific binding. The myoblasts were incubated overnight at 4 °C with a mouse anti-PCNA primary antibody (sc-56 - Santa Cruz, USA) diluted in 1% BSA (1:500). The cells were washed and then incubated for 90 minutes at room temperature with an anti-mouse HRP secondary antibody (ab6789 - Abcam, USA) diluted in 1% BSA (1:500). After an additional washing step, the myoblasts were incubated with DAB solution (500 μL 3,3′-Diaminobenzidine, 4 mL hydrogen peroxide and 4.5 mL PBS - Sigma-Aldrich, USA) to identify proliferative cells expressing PCNA. Hematoxylin was used to counterstain the myoblasts, and the coverslips were dehydrated in a graded alcohol series and mounted with Permount. Proliferation was quantified as a percentage of PCNA-positive cells of the total number of nuclei in 10 images per coverslip using ImageJ® software^63^. The images were acquired under a light microscope coupled to the digital camera Leica DMC2900 (Leica, Germany).

### Myoblast migration assay

Myoblast migration was assessed by a wound healing assay, which was performed after reaching 80–100% confluency (day 4) in 6-well plates using duplicates from three independent cell cultures. Initially, the cell monolayers were mechanically scratched (“wound”) in the shape of a cross by a 200 µL sterile tip in the center of each well. The cell debris was removed by two washes with PBS, and the myoblasts were incubated in DMEM media with 1% antibiotics and 2% fetal bovine serum (Sigma-Aldrich, USA) to reduce the cell proliferation rate. The wound areas were evaluated at 0, 6, 12, 18, 24, 30 and 48 hours, and images were captured under an inverted microscope coupled to the digital camera AxioCam ICc5 (Zeiss, Germany). The wound areas corresponding to the area mean of the four lines in the cross-shaped wound were measured using ImageJ® software^63^. The wound areas were used to calculate the reduction in the wound area size over time (wound closure) and the variation in wound closure over the time interval (migration rate or migration speed).

### Superoxide dismutase and catalase activity

The assays used to verify the activities of antioxidant enzymes were performed after reaching 80–100% confluency (day 4) in 6-well plates using duplicates from three independent cell cultures. The protein content was extracted from the myoblasts using RIPA buffer and a protease inhibitor cocktail (Sigma-Aldrich, USA), which is consistent with the manufacturer’s recommendations. The extracted protein content was quantified by the Bradford method^64^ and used to evaluate the activities of the antioxidant enzymes SOD and CAT. The activity of the SOD enzyme was determined by its ability to inhibit the reduction in nitroblue tetrazolium, causing changes in the color intensity. SOD activity was measured in proper solution (50 mM PBS pH 7.4, 0.1 mM EDTA, 62 μM nitroblue tetrazolium, 98 μM NADH and 3.3 μM phenazine methosulfate - Sigma-Aldrich, USA), and the SOD unit was defined as the amount of enzyme needed to decrease the reference rate to 50% of maximum inhibition^65^. The activity of the CAT enzyme was determined by its ability to decompose hydrogen peroxide (H_2_O_2_) and was also measured in proper solution (10 mM H_2_O_2_ and 50 mM sodium and potassium phosphate buffer pH 7.0 - Sigma-Aldrich, USA). The CAT unit was defined as the amount of enzyme necessary for H_2_O_2_ decomposition at a constant rate of 15 seconds at 240 nm^66^. The enzyme activities were analyzed using the EON microplate reader system with Gen5 2.0 Software (BioTek Instruments, USA).

### mRNA expression

The expression analyses were performed after 80–100% confluence (day 4) in 6-well plates using duplicates from four independent cell cultures. The total RNA was extracted from the myoblast cell cultures using TRIzol® Reagent (Thermo Fisher Scientific, USA), according to the manufacturer’s recommendations. The RNA was quantified using a NanoVue™ Plus spectrophotometer (GE Healthcare, United Kingdom), and the estimation of the RNA purity was performed by measuring the absorbance at 260 nm (RNA quantity) and 280 nm (protein quantity). The RNA integrity was evaluated through capillary electrophoresis in the 2100 Bioanalyzer (Agilent, USA), which provided a RNA integrity number (RIN) based on the *28S* and *18S ribosomal RNAs*. Only samples with 260/280 ratio ≥1.8 and with an RIN ≥7.0 were used. To eliminate any possible contaminating genomic DNA from the samples, the extracted RNA was treated with DNase I, Amplification Grade (Thermo Fisher Scientific, USA). The RNA reverse transcription was performed using the High Capacity cDNA Archive Kit (Thermo Fisher Scientific, USA), according to the manufacturer’s guidelines.

Primers for the *myod1* (*myogenic differentiation 1*), *myog* (*myogenin*), *igf1* (*insulin like growth factor 1*), *mtor* (*mechanistic target of rapamycin*), *fbxo32* (*f-box protein 32*), *rpl13* (*ribosomal protein L13*), *ppiaa* (*peptidylprolyl isomerase Aa*) and *gapdh* (*glyceraldehyde-3-phosphate dehydrogenase*) mRNAs were designed from *Piaractus mesopotamicus* transcriptome, obtained by our group^45^ and whose raw reads were deposited in the European Nucleotide Archive - ENA (accession number PRJEB6656). These primers were designed using *Primer3 v*.*0*.*4*.*0*^67,68^ and *NetPrimer* (Premier Biosoft, USA) softwares (Table 1).

**Table 1:** Primers used for *myod1*, *myog*, *igf1*, *mtor*, *fbxo32*, *rpl13*, *ppiaa* and *gapdh* mRNA amplification by qPCR.

Gene	Accession code	Primer (5′-3′)	Amplicon size (bp)
*myod1*	comp144727_c1_seq4	F: GTTCGTCGTCTTCCTCTTGC R: ACCCGTGCTTTAACACCAAC	191
*myog*	comp137034_c4_seq6	F: CAGACCAGAGGTTTTATGAA R: TAGATGTTGGGGATGGCTTG	171
*igf1*	comp134662_c2_seq2	F: ATTCAGCAAGCCAACAGGT R: CGCACAATACATCTCAAGTCG	116
*mtor*	comp141640_c0_seq15	F: TTGGGAGAGACGTACTGC R: CACAGGACTGGTGTAGGAA	145
*fbxo32*	comp145335_c1_seq5	F: TCTTTGGTGCTCCCCTTGTG R: TAAAACCGAGGACGGCTGG	231
*rpl13*	comp141862_c0_seq1	F: ATCAACAGGAAAGTAGCCC R: AGGATGAGTTTGGAGCGGTA	122
*ppiaa*	comp145566_c0_seq1	F: ATTGTGGTTCGTGAAGTCGC R: CCGCTGGGCAGAGTGATTAT	168
*gapdh*	comp140533_c0_seq1	F: ACACACGACGACAAGACCAA R: GTCCCTCTCGCTGAAAACTG	267

F, forward; R, reverse. Gene are as follow: *myod1 (myogenic differentiation 1), myog (myogenin), igf1 (insulin like growth factor 1), mtor (mechanistic target of rapamycin), fbxo32 (f-box protein 32), rpl13 (ribosomal protein L13), ppiaa (peptidylprolyl isomerase Aa) and gapdh (glyceraldehyde-3-phosphate dehydrogenase)*.

The mRNAs expression levels were detected by quantitative real-time PCR (qPCR) using the QuantStudio™ 12 K Flex Real-Time PCR System (Thermo Fisher Scientific, USA). All qPCR performed were compliant with the Minimum Information for Publication of Quantitative Real-Time PCR experiments (MIQE) guidelines^69^. The cDNA samples were amplified using the GoTaq® qPCR Master Mix (Promega, USA) and the respective primers (Table 1), which were synthesized by Invitrogen (USA). The reactions were performed at 95 °C for 10 minutes followed by 40 cycles of denaturation at 95 °C for 15 seconds and annealing/extension at 60 °C for 1 minute. The reaction efficiency was calculated by LinRegPCR software^70,71^, and the specificity of each primer set was evaluated by the dissociation curve at the end of each PCR reaction, which confirmed the presence of a single fluorescence peak. The relative quantification of expression was performed by the 2^−∆∆Ct^ method^72^ using the DataAssist^TM^ v3.01 software (Thermo Fisher Scientific, USA). The mRNA expression levels were normalized to the *rpl13*, *ppiaa* and *gapdh* mRNAs, whose expressions were constant among all samples.

### Statistical analyses

The statistical analyses were performed using the parametric one-way ANOVA test, followed by Tukey’s multiple comparisons test. The data are presented as the mean ± SD of duplicates from three or four independent cell cultures. The statistical significance was set at 5% (p < 0.05) (GraphPad Prism 5 Software, USA).

## Supplementary information

Supplementary information

## Data Availability

All data generated or analysed during this study are included in this published article.
