# Supplementary material for: Ascorbic acid stimulates the in vitro myoblast proliferation and migration of pacu (Piaractus mesopotamicus)
Source: Sci Rep. 2019 Feb 18;9:2229. doi: 10.1038/s41598-019-38536-4 (PMC6379551; doi:10.1038/s41598-019-38536-4)
Supplement: Supplementary file 1 — Supplementary information [file 41598_2019_38536_MOESM1_ESM.pdf]

Ascorbic acid stimulates the *in vitro* myoblast proliferation and migration of pacu (*Piaractus mesopotamicus*).

Bruno Oliveira Silva Duran[1], Guilherme Alcarás Góes[1], Bruna Tereza Thomazini Zanella[1], Paula Paccielli Freire[1], Jessica Silvino Valente[1], Rondinelle Artur Simões Salomão[2], Ana Fernandes[3], Edson Assunção Mareco[2], Robson Francisco Carvalho[1] and Maeli Dal-Pai-Silva[1*]

[1]São Paulo State University (UNESP), Institute of Biosciences, Department of Morphology, Botucatu, São Paulo, Brazil
[2]University of West São Paulo (UNOESTE), Presidente Prudente, São Paulo, Brazil
[3]São Paulo State University (UNESP), Institute of Biosciences, Department of Chemistry and Biochemistry, Botucatu, São Paulo, Brazil

* Corresponding author
E-mail: maeli@ibb.unesp.br

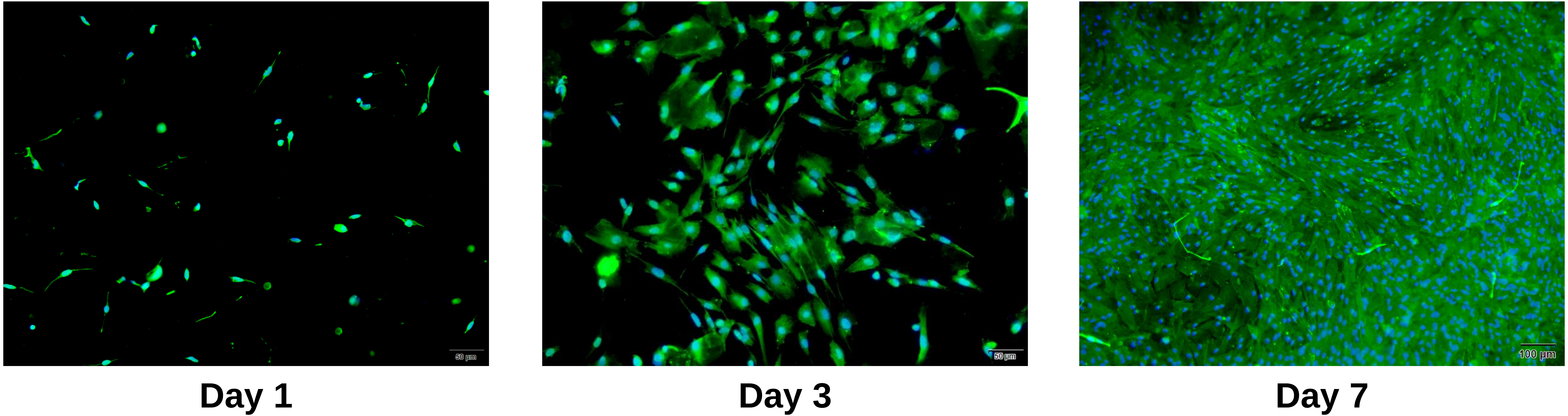

Supplementary Fig S1. Immunofluorescence of myoblast cell cultures established using fast-twitch skeletal muscle from juvenile pacus. The myogenic cells were identified by immunostaining for desmin (green) and the cells nuclei were counterstained with DAPI (blue) on days 1, 3 and 7 of cell culture. The images were obtained under a fluorescence microscope (days 1 and 3 - 20x magnification; Bars: 50 µm; day 7 - 10x magnification; Bar: 100 µm).

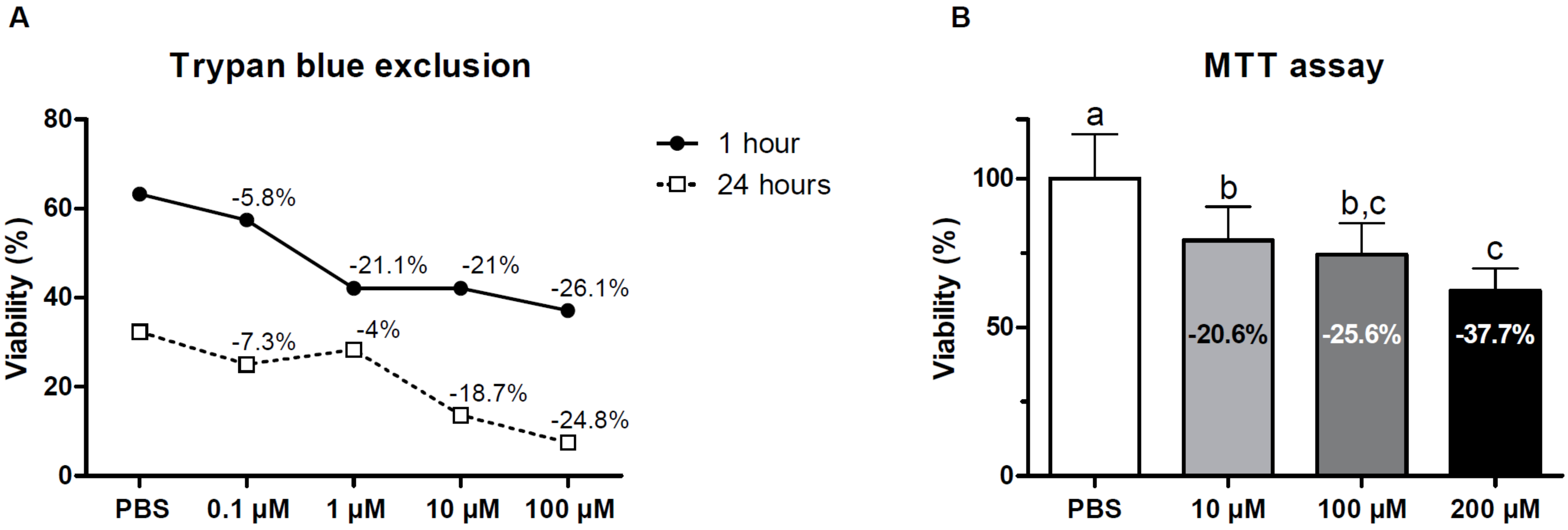

Supplementary Fig S2. The effect of menadione treatment on myoblast viability. (A) Trypan blue exclusion assay. Myoblasts were incubated with PBS or menadione at 0.1, 1, 10 and 100 µM for 1 and 24 hours. The stained (dead) and unstained (live) myoblasts were counted and cell viability expressed as a percentage of the total number of cells. (B) MTT assay. Myoblasts were incubated with PBS or menadione at 10, 100 and 200 µM for 1 hour. The data are expressed as a percentage and presented as the mean ± SD of duplicates from four independent cell cultures. The different letters indicate significant differences among the groups ($p<0.05$ - One-way ANOVA test, followed by Tukey's multiple comparisons test).

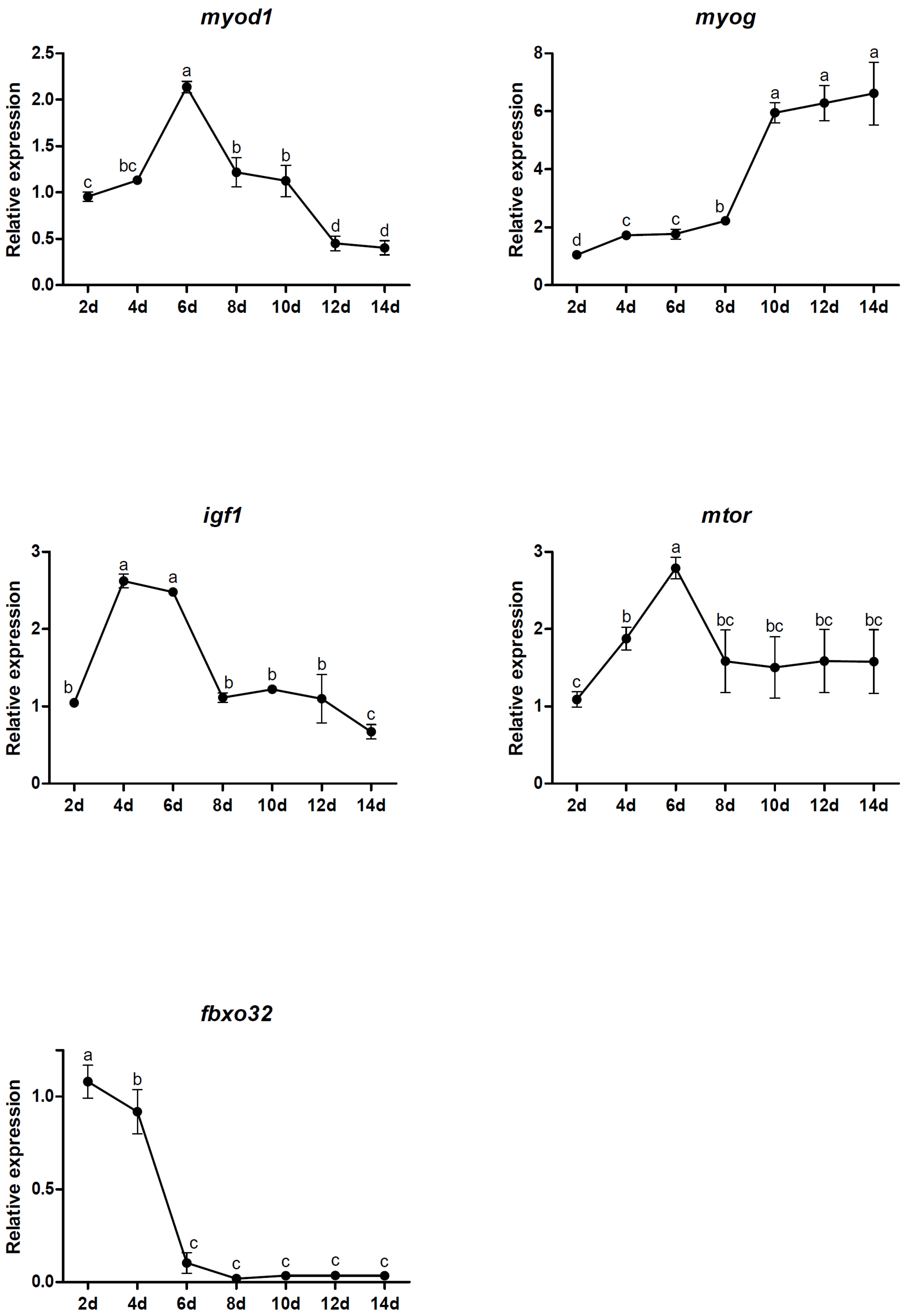

Supplementary Fig S3. Relative mRNA expression of *myod1*, *myog*, *igf1*, *mtor* and *fbxo32* through cell culture development. *myod1*, *myog*, *igf1*, *mtor* and *fbxo32* mRNA expression was assessed by qPCR in myoblast cell cultures on days 2, 4, 6, 8, 10, 12 and 14 of cell culture (d=days). The data are expressed as the fold change compared with 2d and presented as the mean ± SD of duplicates from three independent cell cultures. The different letters indicate significant differences among the groups ($p<0.05$ - One-way ANOVA test, followed by Tukey's multiple comparisons test).
